# ApoA-I Infusion Therapies Following Acute Coronary Syndrome: Past, Present, and Future

**DOI:** 10.1007/s11883-022-01025-7

**Published:** 2022-05-07

**Authors:** Arzu Kalayci, C. Michael Gibson, Paul M. Ridker, Samuel D. Wright, Bronwyn A. Kingwell, Serge Korjian, Gerald Chi, Jane J. Lee, Pierluigi Tricoci, S. Hassan Kazmi, Clara Fitzgerald, Alka Shaunik, Gail Berman, Danielle Duffy, Peter Libby

**Affiliations:** 1 Division of Cardiovascular Medicine, Department of Medicine, Beth Israel Deaconess Medical Center, Harvard Medical School, Boston, MA USA; 2 Baim Institute for Clinical Research, Boston, MA USA; 3 Center for Cardiovascular Disease Prevention, Brigham and Women’s Hospital, Harvard Medical School, Boston, MA USA; 4 CSL Behring, King of Prussia, PA USA; 5 CSL Ltd, Bio21 Institute, Parkville, Australia; 6 Paratek Pharmaceuticals, King of Prussia, PA USA; 7 Division of Cardiovascular Medicine, Department of Medicine, Brigham and Women’s Hospital, Harvard Medical School, 75 Francis Street, Boston, MA 02115 USA

**Keywords:** ApoA-I infusion therapies, Cholesterol efflux capacity, Acute coronary syndrome

## Abstract

**Purpose of Review:**

The elevated adverse cardiovascular event rate among patients with low high-density lipoprotein cholesterol (HDL-C) formed the basis for the hypothesis that elevating HDL-C would reduce those events. Attempts to raise endogenous HDL-C levels, however, have consistently failed to show improvements in cardiovascular outcomes. However, steady-state HDL-C concentration does not reflect the function of this complex family of particles. Indeed, HDL functions correlate only weakly with serum HDL-C concentration. Thus, the field has pivoted from simply raising the quantity of HDL-C to a focus on improving the putative anti-atherosclerotic functions of HDL particles. Such functions include the ability of HDL to promote the efflux of cholesterol from cholesterol-laden macrophages. Apolipoprotein A-I (apoA-I), the signature apoprotein of HDL, may facilitate the removal of cholesterol from atherosclerotic plaque, reduce the lesional lipid content and might thus stabilize vulnerable plaques, thereby reducing the risk of cardiac events. Infusion of preparations of apoA-I may improve cholesterol efflux capacity (CEC). This review summarizes the development of apoA-I therapies, compares their structural and functional properties and discusses the findings of previous studies including their limitations, and how CSL112, currently being tested in a phase III trial, may overcome these challenges.

**Recent Findings:**

Three major ApoA-I-based approaches (MDCO-216, CER-001, and CSL111/CSL112) have aimed to enhance reverse cholesterol transport. These three therapies differ considerably in both lipid and protein composition. MDCO-216 contains recombinant ApoA-I Milano, CER-001 contains recombinant wild-type human ApoA-I, and CSL111/CSL112 contains native ApoA-I isolated from human plasma. Two of the three agents studied to date (apoA-1 Milano and CER-001) have undergone evaluation by intravascular ultrasound imaging, a technique that gauges lesion volume well but does not assess other important variables that may relate to clinical outcomes. ApoA-1 Milano and CER-001 reduce lecithin-cholesterol acyltransferase (LCAT) activity, potentially impairing the function of HDL in reverse cholesterol transport. Furthermore, apoA-I Milano can compete with and alter the function of the recipient’s endogenous apoA-I. In contrast to these agents, CSL112, a particle formulated using human plasma apoA-I and phosphatidylcholine, increases LCAT activity and does not lead to the malfunction of endogenous apoA-I. CSL112 robustly increases cholesterol efflux, promotes reverse cholesterol transport, and now is being tested in a phase III clinical trial.

**Summary:**

Phase II-b studies of MDCO-216 and CER-001 failed to produce a significant reduction in coronary plaque volume as assessed by IVUS. However, the investigation to determine whether the direct infusion of a reconstituted apoA-I reduces post-myocardial infarction coronary events is being tested using CSL112, which is dosed at a higher level than MDCO-216 and CER-001 and has more favorable pharmacodynamics.

## Introductıon

Available therapies effectively reduce low-density lipoprotein cholesterol (LDL-C) levels. Yet, even with very well-controlled LDL-C concentrations, the residual burden of cardiovascular disease (CVD) remains substantial [1]. To reduce this residual risk of CVD, research has also focused on HDL-C as a potential therapeutic target. Several prospective epidemiologic studies, starting with the investigations of Gofman et al. [2] (1966) and Gordon et al. [3] (1977) in the Framingham Heart Study among many others, have demonstrated a consistent and robust inverse relationship between HDL-C levels and cardiovascular risk, irrespective of LDL-C levels or treatment with statins [4, 5, 6]. Furthermore, low HDL-C concentrations correlated with long-term adverse outcomes and mortality in patients with the acute coronary syndrome (ACS) [7, 8]. Meta-analyses have also demonstrated an inverse association of lower HDL-C with an increased cardiovascular (CV) risk among patients treated with statins [5, 9, 10].

These relationships gave rise to the “HDL hypothesis,” which states that “a reduction in plasma HDL may impair the efflux of cholesterol from the arterial wall and thereby accelerate the development of atherosclerosis.” [11, 12] Extensive research has explored various HDL-raising agents with the goal of further reducing cardiovascular risk. The “HDL hypothesis,” however, has encountered recent challenges based on the results of human genetic studies and the null results of several clinical trials that raised HDL-C by administration of fibrates, nicotinic acid (niacin), or various cholesteryl ester transfer protein (CETP) inhibitors.

Yet, strong experimental evidence indicates that HDL particles can mediate reverse cholesterol transport (RCT). The vasoprotective properties of HDL depend strongly on its protein composition, which undergoes substantial alterations and modifications in patients with coronary artery disease [13, 14]. ApoA-I is the principal protein component of HDL and mediates RCT and activation of LCAT, which can affect multiple steps in the RCT pathway. LCAT on HDL particles esterifies free cholesterol and both reduces free cholesterol on the particle surface and favors the accumulation of cholesteryl ester in the core. These actions enable the HDL to both acquire more free cholesterol and deliver more to the liver. Two previous infusible apoA-I compounds did not achieve a significant change in plaque volume in patients with coronary artery disease as assessed by intravascular ultrasound (IVUS). But these approaches had potential limitations mentioned below.

### HDL-C as a Therapeutic Target

#### Fibrates

While fibrates increase HDL-C and lower triglyceride levels, their effectiveness has met with mixed results with respect to cardiovascular event reduction. Although gemfibrozil therapy raised HDL-C and lowered triglycerides in patients with CVD and low HDL-C, the reduction in events did not become apparent until approximately 2 years after randomization [15]. Moreover, the major studies with gemfibrozil (Helsinki Heart Study [16] and VA-HIT [15]) did not specify statins as background therapy. Clinically important drug interaction safety issues limit the use of gemfibrozil combined with statins [17]. In FIELD, fenofibrate did not demonstrate a significant reduction in the risk of coronary heart disease, death, or nonfatal myocardial infarction (MI) compared with placebo in patients with type 2 diabetes mellitus [18]. A subsequent study also demonstrated that the combination of fenofibrate and simvastatin did not reduce the rate of fatal cardiovascular events, nonfatal MI, or nonfatal stroke, as compared with simvastatin alone [19]. A large-scale outcome trial that evaluated a novel selective PPAR alpha modulator, pemafibrate, in patients with diabetes, hypertriglyceridemia (> 200 mg/dL), and low HDL (< 40 mg/dL), on a background of statin therapy. The study was recently halted for futility [20].

#### Niacin

Long-term niacin therapy decreases LDL-C and triglycerides and increases HDL-C levels. Niacin decreased the occurrence of MI in the long-term follow-up of the Coronary Drug Project conducted in the pre-statin era [21]. Outcome trials have not demonstrated a significant reduction in cardiovascular events with niacin-statin combinations despite significant increases in HDL-C levels, as compared with a statin-only approach [22, 23]. Furthermore, niacin therapy caused multiple adverse effects including elevated glucose levels among patients with diabetes [24], an increased risk of developing diabetes [25], peptic ulceration, myopathy, skin rash and ulceration, as well as excess bleeding events (mostly gastrointestinal and intracranial), and infections with combination niacin-laropiprant in patients receiving statins. [26]

#### CETP Inhibitors

CETP garnered great interest because of its role in transferring cholesteryl esters from HDL to apolipoprotein B-containing lipoproteins [27, 28]. The brisk rise in HDL-C induced by CETP inhibitors and the decrease in LDL-C levels with most agents offered considerable promise to prevent cardiovascular events. Yet, contrary to expectations, this therapy failed or only very modestly succeeded in clinical trials, either causing an increase in CV events and deaths (torcetrapib [29]) or lacking efficacy in reducing clinical outcomes despite the rise in HDL-C (dalcetrapib [30], evacetrapib [31]). Torcetrapib raises blood pressure, lowers potassium levels, and elevates sodium and bicarbonate levels due to an off-target mineralocorticoid agonist effect [32]. Anacetrapib treatment showed a modest beneficial effect but more likely due to LDL-C lowering than an elevation in HDL-C [33].

Inhibition of CETP activity increases HDL-C levels. However, the main atheroprotective property of HDL may derive from its ability to act as an acceptor of cholesterol from peripheral cells, specifically arterial wall macrophages. CETP inhibitors may interfere with this process by changing the cholesterol efflux capacity of donor cells and by modulating the uptake capacity of HDL particles [28]. Second, LDL, intermediate-density lipoprotein (IDL), and very-low-density lipoprotein (VLDL) contribute to RCT by CETP-mediated receipt of cholesteryl esters from HDL for the subsequent uptake into the liver. The blockage of this pathway may also contribute to the futility of CETP inhibitors. Furthermore, the concept that very high concentrations of HDL-C could actually deliver free cholesterol to macrophages might contribute to the lack of demonstrable cardiovascular benefit in the members of this class of drugs that elevate HDL concentrations substantially [34]. Taken together, the results of clinical trials indicate that raising HDL-C concentrations alone does not suffice to protect against atherosclerotic events in patients receiving statins.

### Moving from Quantity to Quality

The measurement of the cholesterol in HDL alone cannot fully capture the putative antiatherogenic functions of HDL. HDL contains not only cholesterol but its major apolipoprotein (AI) and some > 50 other associated proteins in variable mixes [35, 36, 37, 38] giving rise to great heterogeneity in HDL particles. While increasing the quantity of HDL-C per se has not reduced CVD risk, altering other aspects of HDL particles may hold more promise [39, 40, 41]. Thus, some researchers have focused on validated atheroprotective functions of HDL like CEC rather than circulating HDL-C levels.

### Cholesterol Efflux Capacity as a Therapeutic Target

**Fig. 1:**
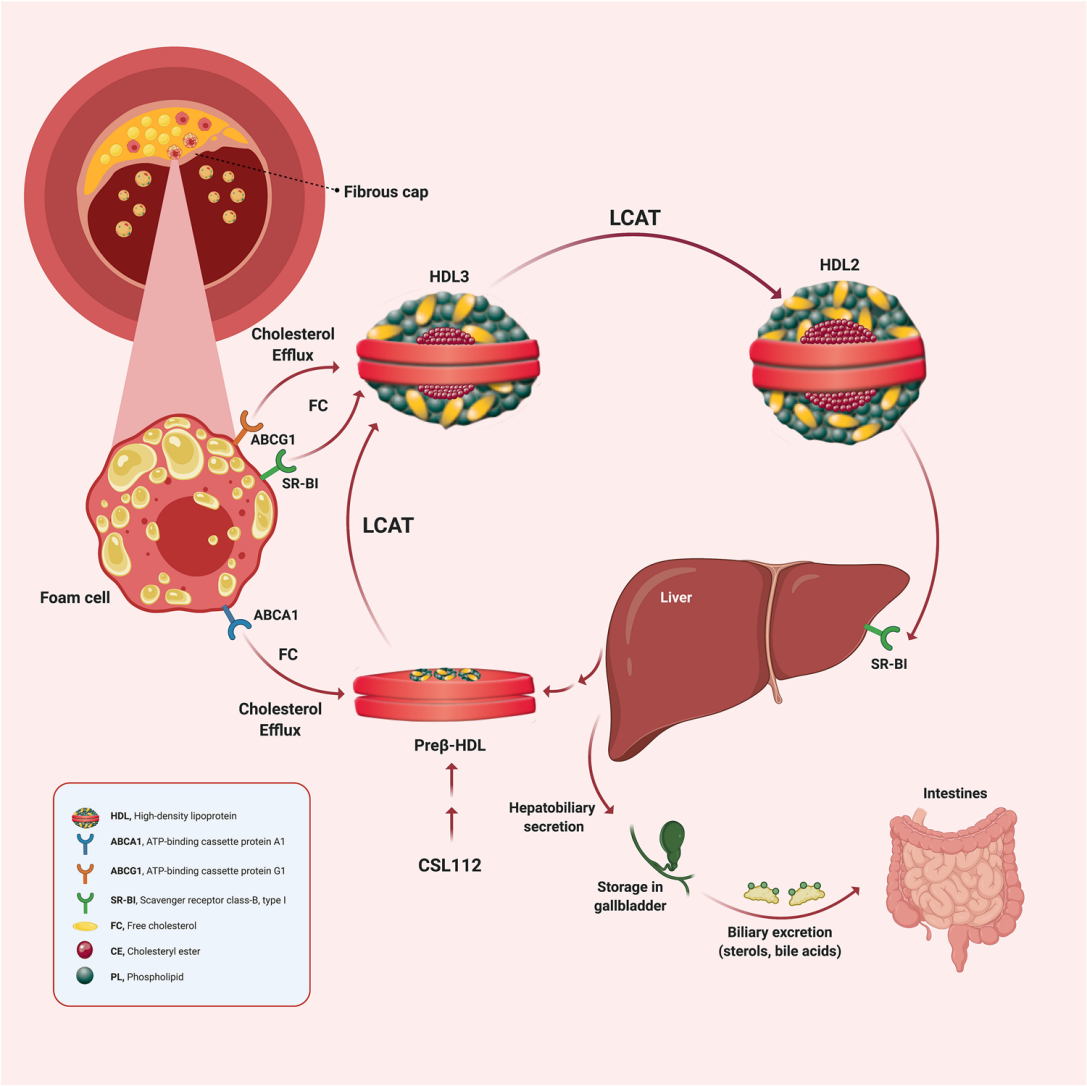
Schematic overview of reverse cholesterol transport. ^†^Free cholesterol in peripheral tissues is effluxed by ABCA1 and ABCG1 transporters to lipid-poor ApoA-I (Preβ-HDL) and larger spherical HDL particles, respectively. The enzyme LCAT, carried on HDL particles, esterifies the free cholesterol molecules to form cholesteryl esters, which migrate to the core of the HDL particle to form mature HDL particles. Subsequently, mature HDL particles deliver the lipid cargo back to the liver through uptake mediated by the scavenger receptor SR-BI. Finally, cholesterol is converted to bile salts in the liver and secreted into the small intestine. CSL112 is apolipoprotein A-I purified from human plasma and reconstituted with phosphatidylcholine to form lipoprotein particles suitable for infusion. CSL112 fuses with HDL in plasma with subsequent release of lipid-poor apoA-I (pre-beta HDL). Abbreviations: *ABCA1*, ATP-binding cassette protein A1; *ABCG1*, ATP-binding cassette protein G1; *ApoA-1*, apolipoprotein A-I; *HDL*, high-density lipoprotein; *LCAT,* lecithin cholesterol acyltransferase; *SR-BI*, scavenger receptor class-B, type I; *FC*, free cholesterol, *CE*, cholesteryl ester, ^†^Created with BioRender.com

Reverse cholesterol transport affects HDL-mediated transport of cholesterol from peripheral tissues to the liver. Cholesterol efflux (CE), cholesterol esterification, lipoprotein remodeling, and hepatic lipid uptake all contribute to this process (Fig. 1). CE from macrophages is the initial step and can be assessed ex vivo as a measure of the biological functionality of HDL particles [42]. CEC relates inversely with atherosclerosis burden, suggesting that CEC mediates atheroprotection by HDL [43]. Increased CEC also correlates strongly with a reduction in incident cardiovascular events, independent of HDL-C and LDL-C concentration [43, 44, 45]. Therefore, one newer path of investigation has focused on boosting HDL’s ability to efflux cholesterol. This shift reflects a refinement of the “HDL hypothesis” to a “cholesterol efflux hypothesis.”

Plasmas with similar HDL-C levels may differ in their ability to promote cholesterol efflux [46], indicating that the cholesterol content of HDL does not fully reflect antiatherogenic HDL properties. Specifically, a high HDL-C concentration may coexist with a low CEC. Therefore, qualitative changes to HDL function, particularly the ability to increase CEC, may relate directly to an improved CVD outcome [47].

### Apolipoprotein A-I (ApoA-I)

ApoA-I, the major protein within HDL, governs in part its size and shape, removes cholesterol from peripheral cells, activates LCAT, and delivers cholesteryl esters to the liver. Evidence from animal and human studies suggests an important role of apoA-I in many of the antiatherogenic effects attributed to HDL [48, 49, 50]. Various peptides, imitating the amphipathic helices in apoA-I, have undergone testing as therapeutic agents including full-length apoA-I, mutated variants of apoA-I, and apoA-I mimetics such as 4F, 6F, FX-5A, ATI-5261, and ETC-642/MDCO-216 [51]. Phase II/III clinical trials in humans have not evaluated these apoA-I mimetic peptides.

### ApoA-I-Based Infusion Therapies

**Fig. 2:**
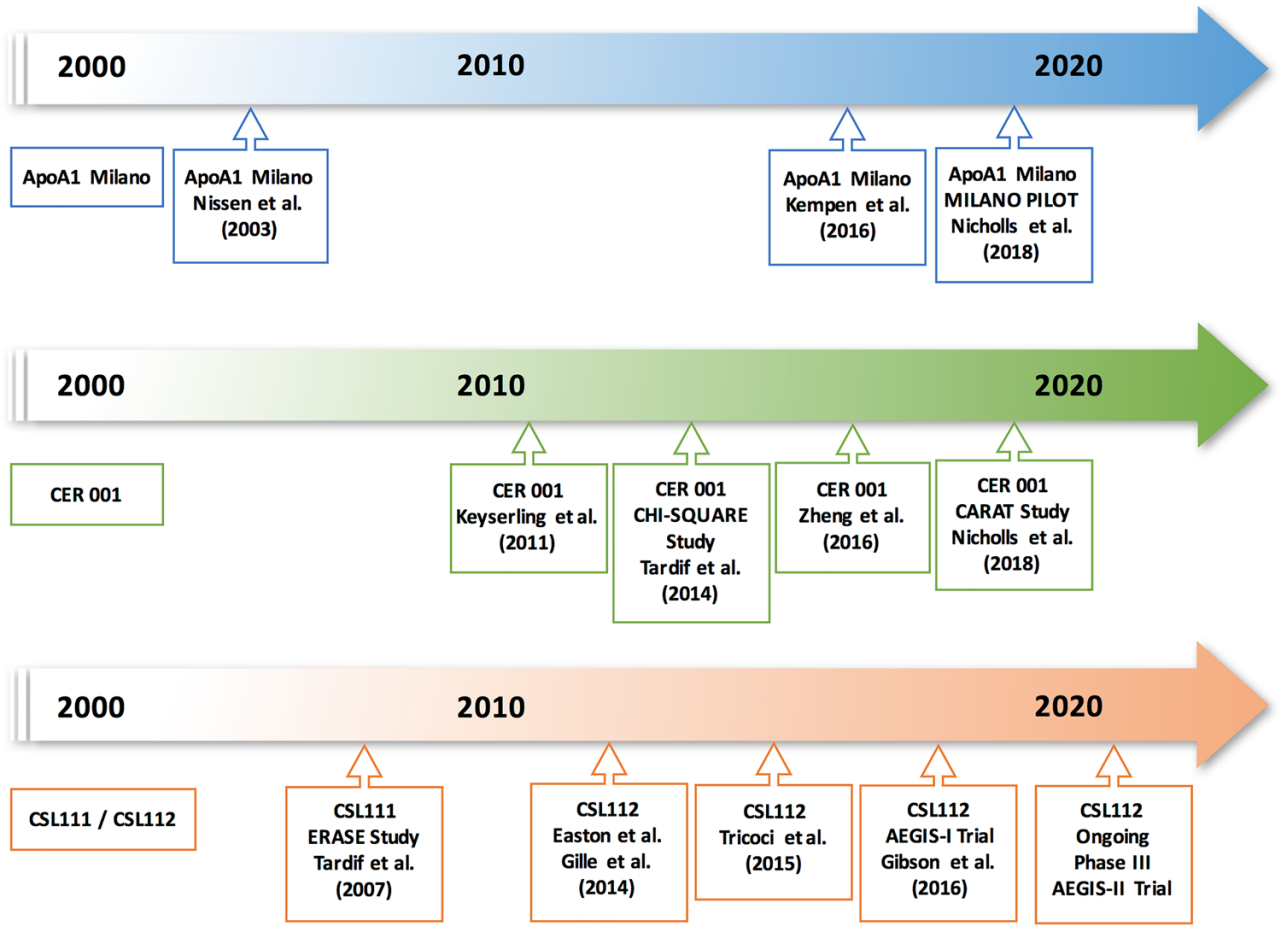
Timeline of human studies on apolipoprotein A-I infusion therapies. ApoA1 Milano (Nissen et al.) [67]; ApoA1 Milano (Kempen et al.) [98]; MILANO PILOT study (ApoA1 Milano, Nicholls et al.) 68.••; CER 001 (Keyserling et al.) [72]; CHI-SQUARE study (CER 001, Tardif et al.) [70]; CER 001 (Zheng et al.) [69]; CARAT study (CER 001, Nicholls et al.) 74.••; ERASE study (CSL111, Tardif et al.) [60]; CSL112 (Easton et al.) [81]; CSL112 (Gille et al.) 80, 82.••••; CSL112 (Tricoci et al.) [104]; AEGIS-I trial (CSL112, Gibson et al.) [83.••••]; AEGIS-II trial design (Gibson et al.) [84]. Abbreviations: *AEGIS-I*, ApoA-I Event Reducing in Ischemic Syndromes I; *AEGIS-II*, ApoA-I Event Reducing in Ischemic Syndromes-II; *ApoA-I*, apolipoprotein A-I; *CARAT*, CER-001 Atherosclerosis Regression Acute Coronary Syndrome Trial; *ERASE*, Effect of HDL on Atherosclerosis-Safety and Efficacy study

Direct infusion of HDL-like particles or transgenic expression of its major proteins may reduce lesion burden in experimental atherosclerosis as well as favorably modify plaque functional features thought to relate to clinical benefit [52, 53, 54, 55]. Such findings in animals led to the hypothesis that infusion of apoA-I containing particles, such as lipid-poor pre-β HDL (the nascent lipid-poor precursor of mature HDL), can render plaques less likely to disrupt and regress atherosclerosis. Subsequently, small human studies have reported favorable effects of HDL-like particle infusion on endothelial vasodilator function and atherosclerosis [56, 57, 58, 59]. The effects of infusing purified forms of HDL on lipid transporting factors, endothelial functions, and changing atheroma volume [60] suggest that administering nascent HDL-like particles is functionally active in promoting cholesterol efflux as opposed to raising HDL-C concentration per se, and may have more relevance to reducing cardiovascular risk [61, 62]. Therefore, engineered HDL particles prepared in vitro from human plasma-derived or recombinant apoA-I and phospholipids merit testing to assess whether their infusion could reduce the burden of coronary atherosclerosis and yield clinical benefit. To date, three infusible HDL mimetics have proceeded from preclinical testing to clinical trials in humans (Fig. 2).

#### ApoA-I Milano

In 1974, University of Milan researchers first described apoA-I Milano. It contains an Arg to Cys mutation at residue 173 and is associated with low HDL-C concentration and modest hypertriglyceridemia but with no apparent elevation of cardiovascular risk [63, 64]. Recombinant apoA-I Milano produced in bacteria and complexed with phospholipids was synthesized and studied with the hypothesis that it would modulate atheroma.

Preclinical studies [65, 66] predicted a potential therapeutic scenario that contemplated an infusion following ACS to augment cholesterol efflux and stabilize plaque, while ongoing conventional lipid-modulating therapy would provide long-term clinical benefits. A phase II clinical trial evaluated 57 subjects following ACS. The subjects received weekly infusions of apoA-I Milano dimer complexed with palmitoyl-oleoylphosphatidylcholine (ETC-216, Esperion Therapeutics) at a dose of 15 or 45 mg/kg for 5 weeks. The investigators used intravascular ultrasonography (IVUS) to compare baseline coronary atherosclerosis to that measured 2 weeks after the final infusion of ETC-216. Plaque volume fell by 4.2% relative to the baseline, but the difference was not significant compared to the placebo arm [67]. Nonetheless, these findings offered promise regarding a novel strategy for the management of ACS.

MILANO-PILOT, a double-blind, randomized, placebo-controlled, multicenter trial evaluated the next iteration of an apoA-I Milano therapeutic, MDCO-216 [68.••]. Post-ACS patients (*n* = 122) on contemporary high-intensity statin therapy received MDCO-216 (20 mg/kg) for 5 weeks. HDL-C levels fell in the MDCO-216 group but not in the placebo group. IVUS showed no significant difference between the treatment and placebo arms in percent atheroma volume, total atheroma volume, and atheroma volume in the most diseased segment.

##### CER 001

CER-001 is an engineered negatively charged lipoprotein particle that contains recombinant human apoA-I derived from cultured cells and a combination of two phospholipids, sphingomyelin, and dipalmitoylphosphatidylglycerol. CER-001 has shown the ability to target plaque regions experimentally and clinically and elevate plasma-free (unesterified) cholesterol (FC) [69]. The “Can HDL Infusions Significantly Quicken Atherosclerosis Regression” (CHI-SQUARE) study [70] investigated the effects of six weekly CER-001 infusions at doses of 3, 6, or 12 mg/kg or placebo in 507 patients with ACS. IVUS and quantitative coronary angiography (QCA) evaluated coronary atherosclerosis at baseline and 3 weeks after the final infusion. CER-001 infusion induced a dose-related increase in blood FC. Yet, compared to placebo, no dose of CER-001 tested produced a statistically significant reduction in either the nominal or the percent total atheroma volume.

Subsequently, a post hoc analysis focusing on anatomically matched arterial segments demonstrated that infusions of CER-001 at a dose of 3 mg/kg provided the greatest atheroma regression in patients with a baseline percent atheroma volume of more than 30% but not with other concentrations of CER-001 [71]. The authors speculated that CER-001 had a U-shaped dose–response curve based on the imaging efficacy results, suggesting higher doses of CER-001 less effectively slowed the progression of atherosclerotic plaque. In this regard, CER-001 dose-dependently increases FC and plasma apo-A1 concentrations [70, 72], with a linear rather than a U-shaped relationship. These IVUS-based results led to the selection of 2–5 mg/kg of CER-001 as the optimal dose [71]. Building on this finding, the “Effect of Serial Infusions of CER-001, a Pre-β High-Density Lipoprotein Mimetic, on Coronary Atherosclerosis in Patients Following Acute Coronary Syndromes in the CER-001 Atherosclerosis Regression Acute Coronary Syndrome” (CARAT) study evaluated the effect of a 3 mg/kg CER-001 dose in 301 post ACS patients with a baseline percent atheroma volume > 30% in the proximal 10 mm in a target vessel [73, 74.••]. Subjects received randomly allocated weekly infusions of either CER-001 or placebo for 10 weeks. Compared to placebo, ten weekly infusions of 3 mg/kg CER-001 did not significantly affect coronary disease progression (percent atheroma volume and total atheroma volume) as assessed by IVUS. The negative results of infusing CER-001 in the setting of contemporary statin therapy in patients with recent ACS did not encourage the further development of this therapy.

#### CSL111 and CSL112

CSL111 combines apoA-I isolated from human plasma with phosphatidylcholine derived from soybean. The randomized, double-blind, placebo-controlled “Effect of HDL on Atherosclerosis-Safety and Efficacy” (ERASE) study used imaging to test CSL111’s actions [60]. One hundred and eighty-three patients received 4 weekly infusions of either placebo or 40 mg/kg of CSL111, starting within 2 weeks of ACS. The investigators performed IVUS and a quantitative coronary angiography (QCA) before randomization and 2 weeks after the last infusion. Atheroma volume fell significantly compared to baseline, similar to the ApoA-I Milano Trial. The therapy did not however promote a significant overall reduction in plaque volume when compared with a placebo.

Nevertheless, CSL111 had favorable effects on plaque characterization index on IVUS and coronary score on QCA [60]. Plaque characterization index is calculated using a detailed analysis of plaque composition for each IVUS cross-section chosen for the analysis at both baselines and after treatment. Every chosen cross-section was divided into 5 regions according to the type of plaque consisting calcific, fibrotic, fibrohypoechoic, hypoechoic, and normal. Plaque characterization scores including arc, area, inner perimeter, and outer perimeter were calculated by means of weighting factors. The summation of the characterization scores from each chosen cross-section was divided by the number of cross-sections analyzed [75, 76]. From a safety perspective, the highest dose of CSL111 was associated with transaminase elevations, which led to the early discontinuation of the 80 mg/kg study group. The hepatic side effect of CSL111 may have arisen from the high phosphatidylcholine content of the particle [77].

CSL112, a second-generation formulation, aims to optimize cholesterol efflux via ATP-binding cassette transporter A1 (ABCA1), a cholesterol transporter induced by excess cellular cholesterol [78] and present in atherosclerotic plaque [79]. CSL112 consists of purified human apoA-I combined with a lower amount of phosphatidylcholine than CSL111. CSL112 first underwent evaluation in a single ascending dose (SAD) study [80] in 57 healthy individuals and was well tolerated over the dose range of 5 to 135 mg/kg. Subsequently, a multiple ascending dose (MAD) study examined 36 healthy subjects within four treatment groups [81]. Groups 1 and 2 received 4 weekly infusions of either low (3.4 g) or high-dose (6.8 g) CSL112, group 3 received the low dose of CSL112 twice weekly for 4 weeks, and group 4 received a placebo. The infusion of 6.8 g rapidly produced a 17-fold increase in pre-β HDL levels [82.••••]. This immediate rise correlated with incremental ABCA1-mediated cholesterol efflux measured in vitro. The multiple infusions of CSL112 were well tolerated and did not cause clinically relevant elevations of liver function indices or evidence of anti-drug antibodies.

The AEGIS-I trial [83.••••] (ApoA-I Event Reducing in Ischemic Syndromes I), a multicenter, randomized, placebo-controlled, dose-ranging phase 2b clinical trial, examined the safety and tolerability of CSL112 compared with placebo. The study included 1258 patients following an acute MI. Four weekly administrations of CSL112 at both low (2 g) and high dose (6 g) were well tolerated and did not lead to significant alterations in the liver or kidney function or other safety concerns. Furthermore, this study confirmed the ability of CSL112 treatment to enhance cholesterol efflux capacity in vitro (3.67-fold for the 2 g dose, 4.30-fold for the 6 g dose).

The ApoA-I Event Reducing in Ischemic Syndromes-II (AEGIS-II) trial (NCT03473223) is investigating the potential of CSL112 to reduce major adverse cardiovascular events among patients with recent AMI. This phase III, a multicenter, double-blind, randomized, placebo-controlled, parallel-group trial is comparing 4 weekly infusions of 6 g CSL112 versus placebo in approximately 17,400 subjects [84].

## Discussion

Although population-based epidemiological studies have consistently demonstrated an inverse relationship between HDL-C concentration and cardiovascular events, clinical trials have reported no or modest improvements in clinical outcomes from therapies that raise endogenous HDL-C. Following the disappointing results associated with HDL-C raising therapies [15, 17, 19, 21, 22, 23, 24, 25, 26, 27, 28, 29], which did not or only modestly improved CEC, the field moved on to assess apoA-I infusion strategies guided by the observation that these intravenous agents may in contrast improve CEC, a strong predictor for cardiovascular events, independent of blood HDL-C concentrations [39, 40].

### IVUS-Based Surrogate Endpoints in Clinical Trials of ApoA-I Infusion Therapies

Although apoA-I infusion studies have suggested reductions in plaque burden assessed by IVUS when compared to baseline, these studies did not demonstrate a significant difference compared to placebo [60, 67, 69, 70, 74.••]. However, these negative imaging studies do not necessarily indicate that apoA-I-based therapies cannot contribute to cardiovascular event reduction. The evaluation of short-term atherosclerotic volume regression by IVUS may not exclude a reduction in cardiovascular events following apoA-I infusion, since these studies only measured plaque volumes within 6–9 weeks after the index event and plaque volume alone may not reflect numerous functional characteristics of plaques implicated in their propensity to provoke clinical events [85]. Statin trials assessed IVUS outcomes later than the HDL infusion studies, at a minimum of 6 months and generally up to 24 months [86, 87]. Indeed, when evaluated between 8 and 12 weeks after initiation of therapy, the statin IVUS trials did not reveal any significant changes in total atheroma volume or percentage of atheroma volume [88.••].

Elevation in CEC associates with an increase in fibrous cap thickness visualized using optical coherence tomography (OCT) [88.••], a crucial morphological change compatible with rendering plaques less susceptible to rupture. Although the concept of “plaque stabilization” generally considers cap structure and lipid pool size [89, 90], it should also encompass many other plaque properties such as the local inflammatory status, the functions of vascular smooth muscle cells, which synthesize structurally important interstitial collagens that confer fibrous cap strength, and activity of matrix-degrading proteinases, each of which may influence the biomechanical properties of the cap. [85, 91, 92] Intravascular ultrasound does not interrogate these important aspects of atheroma. Experimentally, apoA-I rapidly promotes reverse cholesterol transport in vivo and reduces macrophage number and cholesterol content, as well as increases collagen without any significant change in plaque volume [93]. These features may render plaques less likely to rupture.

The strategy of apoA-I-based infusion therapies has derived only modest support from surrogate endpoint data and has suffered from uncertainty with respect to incompletely validated biomarkers that may or may not associate with improved clinical outcomes. The modest sample size and surrogate endpoints of these studies may lead to false-negative or false-positive conclusions. An adequately powered phase III clinical outcomes trial furnishes the only strategy to assess definitively the efficacy of HDL infusion therapies in patients post myocardial infarction by monitoring outcomes including CV death, MI, and stroke. Statins generally do not manifest clinical benefit in the first year following initiation, and apoA-I infusions might offer a way to provide more immediate prevention of events during the high-risk period post-MI and awaiting the long-term actions of statins.

### Comparison of Structural and Functional Properties of ApoA-1 Infusion Strategies

The major apoA-I-based infusion agents (MDCO 216, CER-001, CSL111, CSL112) differ substantially in composition, dosing, timing, frequency, pharmacokinetics, and pharmacodynamics, and these differences may account for differences in their effects (Table 1).

**Table 1:** Clinical studies and characteristics of apolipoprotein A-I infusion therapies

	**MDCO-216**	**CER-001**	**CSL112**
ApoA-I source	Recombinant apoA-I Milano	Recombinant wild-type apoA-I	Native apoA-I isolated from human plasma
Phospholipid	POPC	SM and DPPG in a molar ratio of 32.3:1	Mixed PCs isolated from soy
Protein/phospholipid ratio	1:1.1	1:2.7	1:1.4
Phase IIb study	MILANO-PILOT	CARAT	AEGIS-I trial
Tested patient population	Acute coronary syndrome	Acute coronary syndrome	Acute coronary syndrome
Primary outcome	Coronary atherosclerotic plaque regression in the treatment group	Coronary atherosclerotic plaque regression in the treatment group	Hepatic and renal safety and tolerability of CSL112
Dose mg/kg	20	3	80 (est)
Dose (total), g	1.5 (est)	0.225 (est)	6
Timing of first infusion from first medical contact, days	14	14	5
Dose frequency	Weekly	Weekly	Weekly
Number of doses	5	10	4
	**Acute response**		
ApoA-1 level, % change	− 4	+ 6^*^	+ 106
HDL-C level, % change	− 8	NA	+ 26
ABCA1-mediated CEC, % change	+ 80–90	+ 14^*^	+ 242
LCAT activity			

^*^Parameters are not available in the CARAT study; data from Zheng KH et al. [69] 2016

Abbreviations: *ABCA1*, ATP-binding cassette protein A1; *AEGIS-I*, ApoA-I Event Reducing in Ischemic Syndromes I; ApoA-1, apolipoprotein A-I; *CARAT*, CER-001 Atherosclerosis Regression Acute Coronary Syndrome Trial; *CEC*, cholesterol efflux capacity; *DPPG*, dipalmitoylphosphatidylglycerol; *est*, estimated; *HDL-C*, high-density lipoprotein cholesterol; *MILANO-PILOT*, MDCO-216 Infusions Leading to Changes in Atherosclerosis: a Novel Therapy in Development to Improve Cardiovascular Outcomes—Proof of Concept IVUS, Lipids, and Other Surrogate Biomarkers; *NA*, not applicable; *PC*, phosphatidylcholine; *POPC*, 1-palmitoyl-2-oleoylphosphocholine; *SM*, sphingomyelin; *LCAT*, lecithin cholesterol acyltransferase

The components of these agents include variable proteins and lipid moieties. MDCO-216 contains recombinant apoA-I Milano, CER-001 contains recombinant apoA-I, CSL111, and CSL112 contains human plasma-derived apoA-I. Human apoA-I Milano often associates with low apoA-I levels [94]. Individuals heterozygous for the apoA-I Milano mutation have very low plasma apoA-I and HDL cholesterol levels as well as moderately elevated triglycerides [63]. Low levels of apoA-I Milano in plasma result at least in part from reduced LCAT activation [95], which reduces the transfer of cholesterol to the core of HDL particles reducing their size and increasing the clearance rate [96, 97]. Consistent with prior observations, the MILANO-PILOT trial [68.••] demonstrated that 5 weekly infusions of MDCO-216 caused a significant reduction in native apoA-I compared to placebo (*p* = 0.003). The mechanism of this reduction could result from either hypercatabolism similar to the pathway in heterozygous apoA-I Milano mutation or from a decrease in endogenous production of apoA-I.

The loss of native apoA-I due to apoA-I Milano administration may have adverse consequences. Furthermore, a single ascending dose study [98] reported that infusion of MDCO-216 caused a time and dose-dependent decrease in cholesterol esterification rate (CER) likely due to the reduced activity of LCAT. The proposed mechanism for this effect implicates the mutation in apoA-I Milano that affects the domain on apoA-I, which normally binds to and allosterically activates LCAT [99]. Patients receiving MDCO-216 exhibited a rapid increase in plasma FC, while cholesterol levels remained stable in the placebo group. The increase in FC may have resulted from incremental MDCO216-induced scavenger receptor class B, type I (SR-BI)-mediated efflux of cholesterol from tissues into plasma. This effect could also result from hampered esterification of FC by LCAT, since FC significantly correlated inversely with a decrease in cholesterol esterification rate. CER-001 may also inhibit LCAT activity since infusion leads to elevation of plasma FC with no elevation or even a reduction in cholesteryl ester [100]. CER-001 contains sphingomyelin, a lipid that can inhibit LCAT [101]. In contrast to the other HDL infusion therapies, however, infusion of CSL112 increases cholesterol esterification and maintains stable triglyceride levels [82.••••].

### Comparison of Timing

The timing of administering these apoA-I therapies may prove critical because most recurrent events post ACS occur in the early days and weeks following initial presentation. Despite optimal medical therapy, acute MI survivors have a high risk of experiencing another CV event, especially in the first month. Recurrent CV events occur in 12% of these patients within 1 year, and half of those events occur in the first 30 days [102]. Therefore, an optimal intervention would achieve plaque stabilization very early after the initial presentation during a period before statins have achieved their expected clinical benefit. Participants in the CHI-SQUARE, MILANO, and CARAT studies received the first HDL therapy infusion within 14 days post ACS, and the infusions were repeated weekly on 6, 5, and 10 occasions, respectively. To address the high incidence of early recurrent CV events following ACS, AEGIS-II targets an earlier initiation and completion of treatment compared to previous studies. The first infusion of CSL112 will occur within 5 days after the index MI, and the total duration of once-weekly infusions is 4 weeks with an assessment of the primary endpoint at 90 days and follow-up through 1 year to assess the durability of the effect.

### Comparison of Dosing

The dosing of these 3 agents differs over a 30-fold range, indicating the uncertainty in the optimal target dose and the potential for underdosing. The pivotal trials used CER-001 (3 mg/kg) and MDCO-216 (20 mg/kg) based on pilot imaging efficacy outcomes [71, 103]. CSL112 is dosed at a higher total dose of 6 g, corresponding to approximately 80 mg/kg based on its pharmacodynamic response. The higher dose of CSL112 yields a marked increase in plasma apoA-I concentrations and ABCA1-mediated CEC [80, 81, 104]. As a result, CEC differs substantially from these other three therapies. ABCA1-mediated CEC, which might be a relevant CEC measure for apoA-I therapies, increased by 242% in the AEGIS-I trial [83.••••] of CSL112, compared with the 80 to 90% increase with MDCO-216 in the MILANO-PILOT trial [68.••] and the minimal rise of 14% for CER-001 in an earlier study [69]. CARAT did not report CEC levels [74.••].

## Conclusion

Although previous imaging studies of apoA-I infusion therapies did not demonstrate significant regression in coronary atherosclerotic plaque, post MI infusion of CSL112 differs in its properties from the other infusion strategies tested. CSL112 contains a human plasma-derived apoA-I, unlike the recombinant MDCO-216 and CER-001 proteins. Furthermore, CSL112 is not a mutant apoA-I like MDCO-216. CSL112 augments LCAT function in contrast to MDCO-216 and CER-001, which both lower LCAT function. CSL112 does not reduce endogenous apoA-I concentrations as does the MDCO-216 molecule.

Impaired CEC associates with worse clinical outcomes, and CSL112 substantially raises CEC. The effect of CSL112 to elevate concentrations of apoA-I and pre-β HDL results in a marked increase in CEC, as reported in phase I and validated in phase II clinical trials in healthy volunteers [81], patients with stable atherosclerotic coronary vascular disease [104], and after acute myocardial infarction [83.••••]. The ability of CSL112 to elevate strongly CEC suggests that it might rapidly remove plaque cholesterol and reduce the risk of early recurrent cardiovascular events. CSL112 has so far raised no safety concerns. The large phase III AEGIS-II trial will test the potential of early administration of CSL112 to reduce major adverse cardiovascular events in the 90-day high-risk period post-myocardial infarction. This study should provide insight into whether enhancing cholesterol efflux can improve cardiovascular outcomes.

## Abbreviations

ABCA1: ATP-binding cassette transporter protein A1
ACS: Acute coronary syndrome
ApoA-I: Apolipoprotein A-I
CE: Cholesterol efflux
CEC: Cholesterol efflux capacity
CETP: Cholesteryl ester transfer protein
CV: Cardiovascular
CVD: Cardiovascular disease
FC: Free cholesterol
HDL-C: High-density lipoprotein cholesterol
IDL: Intermediate-density lipoprotein
IVUS: Intravascular ultrasonography
LCAT: Lecithin cholesterol acyltransferase
LDL-C: Low-density lipoprotein cholesterol
MI: Myocardial infarction
MACE: Major adverse cardiac events
MAD: Multiple ascending dose
Pre-β HDL: Lipid poor precursor of mature HDL
QCA: Quantitative coronary analysis
RCT: Reverse cholesterol transport
SAD: Single ascending dose
SR-BI: Scavenger receptor class B type I
VLDL: Very low-density lipoprotein
